# Survivorship and (QALY)ty: In Pursuit of a Mirage?

**DOI:** 10.5005/jp-journals-10071-23130

**Published:** 2019-03

**Authors:** Bharath Kumar Tirupakuzhi Vijayaragavan, Nagarajan Ramakrishnan

**Affiliations:** 1,2 Institute of Critical Care Medicine, Apollo Hospitals, Chennai, India

## Abstract

**How to cite this article:** Vijayaragavan BKT, Ramakrishnan N. Survivorship and (QALY)ty: In Pursuit of a Mirage? Indian J Crit Care Med 2019;23(3):111-112.

Post intensive care syndrome (PICS) refers to new or worsening impairments in physical, cognitive or mental health that result from an episode of critical illness and last beyond discharge from the Intensive Care Unit (ICU)^1^. Depending on the component affected, the incidence of PICS varies from 25% for physical and cognitive to as high as 60% for psychiatric manifestations^2^. As we make rapid strides in improving the quality of intensive care globally and as mortality rates decrease, post ICU survivorship has become an increasing focus of research over the past decade. Several studies from high income countries (HICs) have documented the intermediate to long term consequences of surviving an episode of critical illness^3,4,5^.

In this issue, Mishra et al.^6^ report on the results of post ICU follow-up from a large tertiary care public, university hospital in India and attempt to perform a cost effectiveness analysis of the ICU stay. From a survivor cohort of 758 patients, the authors were able to contact 113 patients (15% of the cohort) and obtain follow-up data on quality of life for 86 patients who were alive using the validated SF-36 tool. Interestingly, the authors had to rely on population data from Australia for the comparison on quality of life as similar data from India is lacking. While survivors scored lower on physical function and general health perception, these differences were not statistically significant and overall, patients had a quality of life similar to the general population. When the authors dichotomized by age, the quality adjusted life years (QALY) gained was similar for patients below and above 50 years. The cost per QALY was also similar in this population.

The authors need to be commended for this important study. There is paucity of data from India and from the wider South Asian region on post ICU outcomes and this study provides important insights into the challenges of ICU survivorship. The distinct epidemiology of critical illness in this region i.e., considerable burden of tropical illness, delays in recognition and access to health care, baseline malnutrition and frailty, absence of a social safety net and a predominant out of pocket payer model. There are all mean that the impact of critical illness on intermediate-long term outcomes are likely very different to patients from HICs. The authors have also done well to quantify the cost-effectiveness of intensive care. Cost-effectiveness analysis in the ICU specifically and more generally in health care are typically minefields that few researchers attempt to navigate. In lower middle-income countries (LMICs), these challenges are further amplified by wide variations in health-care delivery and payment models. Several of these issues have been highlighted in a previous review in the journal by Jayaram and Ramakrishnan^7^.

Despite this admirable effort by Mishra et al.^6^, caution is warranted in interpreting these findings. As pointed out by the authors and as is evident from the number of patients that they were able to contact, follow-up is an incredibly hard nut to crack. Only 15% of the patients were contacted and only 86 patients provided follow-up data (just over 10% of the total cohort), all of which speak to the difficulties of connecting with survivors after discharge. It is also unclear from the paper about the timeline of follow-up for these 86 patents. Designing a follow-up study prospectively and planning for a shorter follow-up period (6 months to 1 year) in future studies may alleviate the problem of loss; however, it will also mean that longer term outcomes (beyond 12 months) remain unavailable. It is also important to note that patients providing follow-up data are by definition ‘different’ than those that do not. This could mean a higher motivation level among survivors who are doing well and hence also provide follow-up information. Thus, the finding of no difference in quality of life among survivors needs to be taken with a grain of salt.

Most quality of life measures are subjective self-assessments and the phenomenon of ‘cheated death’ has been reported previously^8^. This is the patient's proclivity to report a higher quality of life due to the perception of having ‘cheated death’ and hence results in a subjectively better estimate of current functional status. This phenomenon may also have contributed to the authors' findings. On a related note, while it is critical to recognize and identify PICS, it is also important to note that the effectiveness of interventions aimed at improving post ICU quality of life remain disappointing^9,10^. In a recent editorial, Vijayaraghavan and colleagues proposed a triaged approach for post ICU follow-up care^11^.

The cost per QALY in this study is US$ 1396 and the authors interpret this as an acceptable estimate. However, the patients in this study come from a large University public hospital where costs are typically lower. Data from INDICAPS^12^ suggests that the bulk of intensive care in India is delivered through private institutions where cost of care is much higher. It is critical to remember that a quarter of India still experiences multi-dimensional poverty (http://www.in.undp.org/content/india/en/home/sustainable-development/successstories/MultiDimesnionalPovertyIndex.html) and that up to 40% of families in ICUs sell assets to pay for healthcare costs^7^. These issues were also previously highlighted by Ramakrishnan in an editorial in this journal^13^.

Notwithstanding these limitations, the authors have brought to light the consequences of critical illness survivorship and provided an estimate of costs per QALY gained. This is important work that needs to be followed up with further multicentric evaluations from India and the broader region. We recommend the creation of a research consortium that could help accelerate coordinated action on these key research questions. The consortium could bring together urban and rural ICUs and private and public ICUs on a common platform to facilitate research that is regionally relevant and context specific.

In conclusion, as mortality rates continue to decline globally with advances in intensive care, post ICU follow-up and improving the quality of life after critical illness have emerged as the next frontier.
